# Empirical evidence for large X-effects in animals with undifferentiated sex chromosomes

**DOI:** 10.1038/srep21029

**Published:** 2016-02-12

**Authors:** Christophe Dufresnes, Tomasz Majtyka, Stuart J. E. Baird, Jörn F. Gerchen, Amaël Borzée, Romain Savary, Maria Ogielska, Nicolas Perrin, Matthias Stöck

**Affiliations:** 1 Department of Ecology and Evolution (DEE), University of Lausanne, Biophore, CH-1015 Lausanne, Switzerland; 2 Department of Evolutionary Biology and Conservation of Vertebrates, Wrocław University, Sienkiewicza 21, 50-335 Wrocław, Poland; 3 Department of Population Biology, Institute of Vertebrate Biology, Academy of Sciences of the Czech Republic, External research facility Studenec, 675 02 Koněšín, Czech Republic; 4 Leibniz-Institute of Freshwater Ecology and Inland Fisheries (IGB), Müggelseedamm 301, D-12587 Berlin, Germany; 5 Laboratory of Behavioral Ecology and Evolution, School of Biological Sciences, Seoul National University, 151-747 Seoul, Republic of Korea

## Abstract

Reproductive isolation is crucial for the process of speciation to progress. Sex chromosomes have been assigned a key role in driving reproductive isolation but empirical evidence from natural population processes has been restricted to organisms with degenerated sex chromosomes such as mammals and birds. Here we report restricted introgression at sex-linked compared to autosomal markers in a hybrid zone between two incipient species of European tree frog, *Hyla arborea* and *H. orientalis*, whose homologous X and Y sex chromosomes are undifferentiated. This large X-effect cannot result from the dominance or faster-X aspects of Haldane’s rule, which are specific to degenerated sex chromosomes, but rather supports a role for faster-heterogametic-sex or faster-male evolutionary processes. Our data suggest a prominent contribution of undifferentiated sex chromosomes to speciation.

Reproductive isolation is crucial for the process of speciation to progress, and biologists have started to learn about the genomics and ecology of evolving reproductive barriers^1^,^2^. Theory^3^ and empirical evidence^4^,^5^ suggest that sex chromosomes play a central role in the evolution of reproductive isolation between incipient species. Two rules based on empirical data imply that sex chromosomes contribute to the build-up of postzygotic isolation, i.e. hybrid fitness decrease, infertility or inviability. First, Haldane’s rule states that, if one sex is absent, rare, or sterile in an interspecific cross, this is usually the heterogametic sex^6^. This pattern is obeyed by the vast majority of animal taxa^7^,^8^,^9^, and evidence has recently been extended to plants^10^. Second, the large X-effect (also known as “Coyne’s rule” or “large Z-effect”) refers to the disproportionally high impact of X or Z chromosomes, compared to autosomes, in driving hybrid dysfunction^11^. Mapping of Quantitative Trait Loci and backcross analyses in *Drosophila* have provided compelling evidence that genetic factors with the largest effect on hybrid sterility tend to be X-linked^12^,^13^,^14^. So far, as for *Drosophila*, empirical evidence for both of these rules has been restricted to outcomes from interspecies crosses^9^,^15^, or in natural settings to organisms with differentiated sex chromosomes, like mammals and birds.

Several non-exclusive hypotheses predominate to account for Haldane’s rule and large-X-effect observations^16^. The dominance hypothesis suggests that, if alleles responsible for Dobzhanski-Muller incompatibilities are partially recessive, they will have a greater impact when located on hemizygous X (or Z) chromosomes, being exposed in the heterogametic sex^17^,^18^. In addition, such effects can accelerate the rate of genetic changes on X- or Z-linked genomic regions (i.e. the faster-X theory)^3^, which can also contribute to Haldane’s and Coyne’s empirical patterns. Namely, under the faster-X theory, exposure of recessive mutations to selection in hemizygotes and reduced effective population size relative to the autosomes are considered the major causes. Alternatively or in complement, the faster-male theory hypothesizes that stronger sexual selection on males would drive faster evolution of male-expressed genes^19^. This would result in more male than female incompatibilities in hybrids^20^,^21^, and can thus explain Haldane’s rule in XY systems. However, it remains unclear whether such incompatibilities accumulate non-randomly on sex chromosomes^14^. Finally, Dobzhanski-Muller incompatibilities may also involve X-Y (or Z-W) epistatic interactions, for example when complementary alleles on conspecific gametologs are required for proper meiotic segregation or sexual differentiation^22^,^23^, therefore having a disproportionate effect in the heterogametic sex (i.e. the faster-heterogametic sex hypothesis).

The relative contribution of sex chromosomes to postzygotic isolation is thus bound to the mechanisms involved. Notably, it is expected to depend on their level of degeneracy: the strong dominance and faster-X effects acting in species with heteromorphic sex chromosomes should not apply when these remain undifferentiated. Accordingly, F_1_ post-zygotic incompatibilities seem generally more severe in interspecies crosses with heteromorphic than with homomorphic^24^ or no sex chromosomes^15^. In clawed frogs, *Xenopus*, which possess homomorphic Z and W chromosomes, sex-reversal experiments have shown hybrid male sterility to depend on phenotypic (male or female) rather than genetic sex (ZZ or ZW), suggesting little responsibility of the sex chromosomes but still important faster-male effects; specifically, higher sensitivity to perturbations of spermatogenesis in a hybrid background^25^. Estimating the contribution of sex-linked genes to postzygotic isolation in taxa with different sex-determining systems and levels of sex-chromosome degeneracy can thus increase our understanding of the underlying mechanisms.

Hybrid zones present natural laboratories that have served to characterize the nature of reproductive barriers in many organisms^26^,^27^, including amphibians^28^,^29^,^30^. Thus, they appear highly suitable to evaluate the role of sex chromosomes. Indeed, patterns of sex chromosome introgression have been documented in organisms with differentiated sex chromosomes such as mammals^31^,^32^,^33^,^34^,^35^, birds^36^,^37^,^38^,^39^,^40^ and insects^41^,^42^. With few exceptions^43^, these studies reported restricted introgression at sex chromosomes, advocating their prominent role in driving speciation. However, it remains empirically unexplored whether this holds for species with homomorphic sex chromosomes, where dominance and faster-X effects are not at play, and whether this translates into differential introgression patterns in natural hybrid zones.

We address this open question in the European tree frogs *Hyla arborea* and *H. orientalis*, which feature undifferentiated sex chromosomes. These two species are non-sister taxa and have diverged approximately since the late Miocene (~5 My)^44^. After the last glaciation they expanded from the Balkan Peninsula to Western Europe (*H. arborea*), and from Asia Minor and Eastern Europe (*H. orientalis*), and now meet in secondary contact zones in several regions of Eastern Europe, with evidence for hybrids from central Poland to northern Greece^44^. In this southern part of their hybrid zone, *H. arborea* and *H. orientalis* exhibit limited mitochondrial and nuclear introgression (~30 km), restricted to few hybrid populations and sharp genomic transitions, in line with advanced reproductive isolation^45^. Both species inherited the same pair of homomorphic XY sex chromosomes from a common ancestor but X and Y gametologs remain genetically undifferentiated due to occasional recombination^46^,^47^,^48^,^49^.

In this study, we analyse patterns of introgression between these two species across their northern hybrid zone in lowland Poland. Postglacial geological history^50^ constrains this contact to be younger than 14 ka. We found strongly restricted introgression at sex-linked loci compared to other parts of the genome, indicating a large X-effect (or “large sex chromosome effect”). Given that dominance and faster-X processes are not at play in *Hyla*, this pattern implies mechanisms like faster-male and/or faster-heterogametic sex processes instead, and suggests that non-degenerate sex chromosomes can also contribute disproportionally to speciation.

## Results

**Figure 1:**
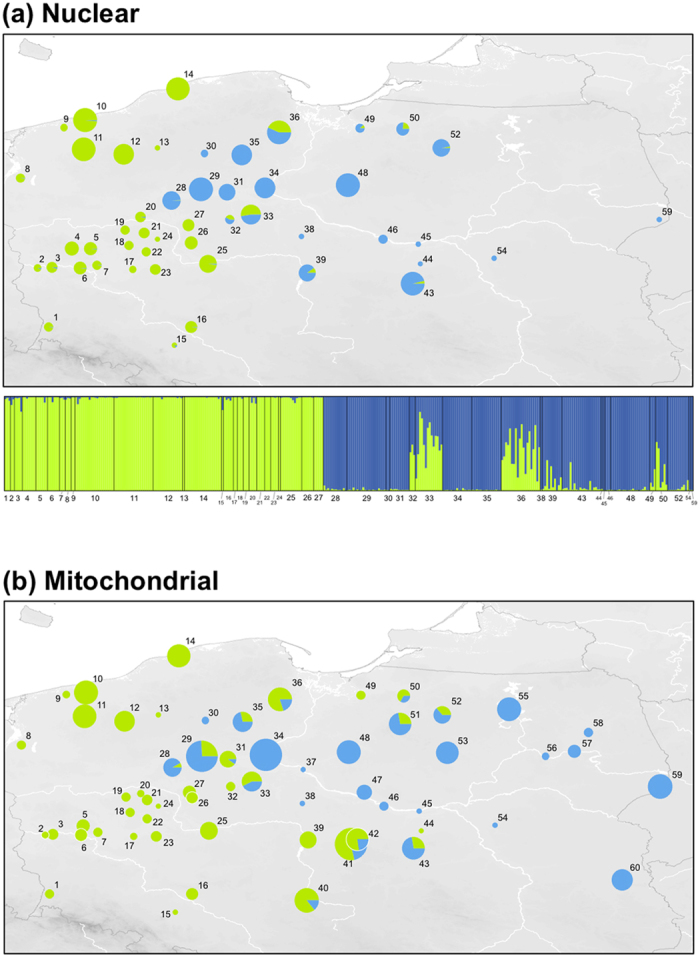
Geographic distribution of nuclear and mitochondrial gene pools in the northern *H. arborea/ H. orientalis* hybrid zone. (**a**) Bayesian clustering assignment of individual microsatellite genotypes by STRUCTURE (barplots) and mean probability of assignment for each population (map); (**b**) Distribution of the mtDNA lineages in populations. Pie charts are proportional to sample sizes. Green: *H. arborea*, blue: *H. orientalis*. Maps were built with ArcGIS 9.3 (ESRI, http://www.esri.com/software/arcgis).

Bayesian assignment of genome-wide individual microsatellite genotypes (29 loci) unambiguously recovered two groups (ΔLL K1→ 2 = 1916.9), corresponding to the gene pools of the two species. This strong two-group signal was confirmed by principal component analysis of individual microsatellite genotypes (File S1). Ancestry coefficients thus correspond to estimates of hybrid index (HI). We found signs of nuclear introgression (intermediate HI estimates) between *H. arborea* and *H. orientalis* over a 200 km wide zone with mosaic contacts and interspersed hybrid populations (e.g. localities 32–33, 36, 50; Fig. 1a) across central and northern Poland. Distribution of mitochondrial haplotypes yielded a similar picture with wider introgression: *H. arborea* mtDNA was frequently sampled further east within the *H. orientalis* range (e.g. localities 39–44, 50–52; Fig. 1b).

**Figure 2:**
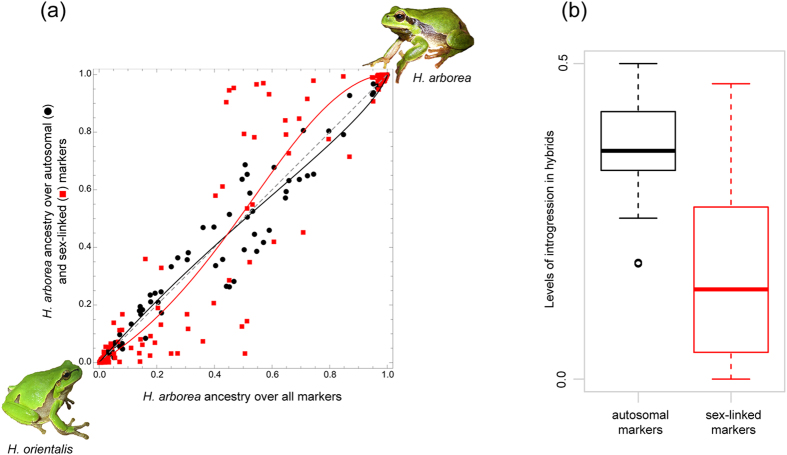
Lower introgression at sex-linked compared to autosomal markers. (**a**) Sex-linked (red squares) and autosomal (black circles) hybrid indices (0: pure *H. orientalis*, 1: pure *H. arborea*) for each individual compared to their HI over all markers. Solid lines are maximum likelihood fits of the data to Fitzpatrick’s genome cline model^51^. Squares show sex-linked and circles autosomal markers. The data is significantly better explained by introducing a separate (steeper) cline for the sex-linked loci (Gtest ΔLL = 4.07, df = 2, *P* = 0.0003; see Results). (**b**) Comparison between sex-linked and autosomal introgression in 32 confirmed hybrids (see Methods); the difference is strongly significant (paired non-parametric Wilcoxon test, p = 4.0 e^−6^). Photo of *H. orientalis*: Matthias Stöck; photo of *H. arborea*: Amaël Borzée.

Comparison of averaged sex-linked vs. autosomal hybrid index (HI) revealed significantly less introgression at sex-linked than at autosomal markers (Fig. 2a). A wide range of hybrid types was sampled, exhibiting intermediate HI estimates for autosomes (i.e. 0 < HI < 1), but showing strongly limited introgression at sex-linked markers (i.e. HI closer to 0 or 1). The likelihood of this data was calculated conditioned on Fitzpatrick’s genome cline model^51^. Specifically, we compared two models, one assuming a single cline fitted to both autosomal and sex chromosomal data, and another involving two separate clines, one fitted to each marker type. The two-clines model had a significantly higher likelihood (Gtest ΔLL = 4.07, df = 2: *P* = 0.0003), strongly supporting reduced introgression of sex chromosome markers. Our inference was robust to three sources of uncertainty. First, 100 replicate STRUCTURE runs led to the same conclusion. Second, the effective sample size^52^ may be overestimated, but the inference remains significant even if the effective size is as low as 37% of the number of alleles sampled. Third, loci can vary in their relative contributions to the HI estimates due to differences in diagnosticity of their alleles, but the inference remained unchanged and significant when locus-specific contributions were considered. Furthermore, the inference signal is also present in subsets of the data: considering only “confirmed” hybrids (n = 32; Methods), the level of introgression significantly differed between autosomal and sex-linked markers (paired non-parametric Wilcoxon test, *P* = 4.0 e^−6^, Fig. 2b).

Additional analyses show that higher estimated admixture at autosomal loci is not caused by a lack of information about their source population: autosomal markers maintain a similar signal of greater admixture irrespective of whether they are analysed on their own or combined with the strong signal of the sex linked loci (Wilcoxon’s *P* = 2.0 e^−10^; File S2a–c). Moreover, to control for the lower statistical power of our sex-linked (n = 8 loci) than of the autosomal (n = 21 loci) marker set, we resampled and analysed 100 subsets of 8 randomly chosen autosomal markers: sex-linked introgression was significantly lower than autosomal introgression in all cases (File S2d). Thus, the lower estimates of admixture at sex-linked compared to autosomal markers in *H. arborea*/*H. orientalis* hybrids reflect real differential introgression rather than differences in marker informativeness.

## Discussion

European (*H. arborea*) and Eastern (*H. orientalis*) tree frogs form a mosaic hybrid zone in the northern parts of their contact across central Poland. Our data show significantly less introgression of sex-linked than of autosomal loci, providing evidence for a “large X-effect”. Applied to homomorphic sex chromosomes, we argue, that this term should rather be modified to “large sex chromosome effect”. Importantly, given homomorphy and occasional X-Y recombination of tree frog sex chromosomes, the dominance or faster-X models of Haldane’s rule, which require degenerate or silenced Y-chromosomes, cannot explain their restricted introgression. In both focal species, X and Y do not show sequence divergence along the chromosomes^46^,^47^,^48^ and in the northern parts of the *H. arborea* range, Y haplotypes have last recombined approximately 15000 years ago^53^, leaving not enough time for Y chromosome decay.

Our findings may rather be explained by faster-male and/or faster heterogametic sex evolution. Male frogs are expected to be under strong sexual selection due to female mate choice, especially in lek-breeding species such as tree frogs^54^. Faster-male effects on hybrid male sterility have been found in pipid frogs (*Xenopus*; see Introduction)^25^. However, this should only constrain sex-chromosome introgression if the genes involved in male sterility disproportionally map to the sex chromosomes, an assumption, which has received little support and which has been rejected for female heterogametic ZW *Xenopus*^25^. In contrast, faster-heterogametic sex effects necessarily imply interactions between X and Y chromosomes. Identifying and mapping genes with male-biased expression and differential introgression in *Hyla* should provide information to estimate the relative contributions of these two processes. Moreover, documenting patterns of meiotic segregation in hybrids might help infer a role for X-Y epistatic interactions. Our results are in line with anecdotal evidence of potential Haldane’s effects reported in a *H. arborea* × *H. orientalis* cross, exhibiting gonadal malformation in a single F_1_ male^49^. At present, we have no information about sex-biased asymmetric assortative mating (e.g. *H. arborea* females preferring *H. orientalis* males but *H. orientalis* females avoiding *H. arborea* males), which could contribute to the differential introgression observed. This seems unlikely, as it would require inverse sex-biased and/or species-biased cross-preferences. It also remains unclear whether sex-biased dispersal, undocumented in *Hyla*, could contribute to the observed pattern.

Our study thus provides the first population genetics evidence that even non-degenerate (homomorphic) sex chromosomes can play a disproportionate role in reproductive isolation between incipient species. It will be interesting to test if this pattern holds in other organisms with homomorphic sex chromosomes, which are widely found among amphibians and fishes, especially given their high diversity of sex-determining systems^55^,^56^. Seeking evidence for Haldane’s rule through experimental crosses may also illuminate the relative importance of mechanisms of speciation^15^. Systems with homomorphic sex chromosomes provide mixed support for Haldane’s rule, dependent on heterogamety: Haldane’s pattern is observed in the majority of male-heterogametic species tested (e.g. newts^57^, teleost fishes^58^,^59^,^60^,^61^,^62^) but evidence is lacking for female-heterogametic species (e.g. *Xenopus*^63^, some bufonid toads^64^, *Populus* trees^65^). If Haldane’s rule mostly arises from dominance and faster-male effects^9^, it should indeed not apply to ZW-systems with homomorphic sex chromosomes. Combining experimental studies of hybrid incompatibilities with population genomic analyses of differential introgression across natural hybrid zones will shed light on the mechanisms underlying postzygotic isolation.

## Methods

### Sampling and DNA extraction

Tree frogs were sampled during the breeding seasons (April-June, 2010–2013) from 60 localities throughout the lowlands of Poland (n = 578 individuals), where *H. arborea* and *H. orientalis* come into secondary contact^44^. DNA was obtained from non-invasive buccal swabs^66^ (adults) or ethanol-fixed tailtips (tadpoles) and extracted using the Qiagen Biosprint Robotic workstation. Details of sampling localities are available in File S3. Tree frog sampling was permitted by the General Directorate for Environmental Protection of Poland (Generalna Dyrekcja Ochrony Środowiska; No. DOP-oz.6401.02.28.2013.JRO). The collection of genetic material from tadpoles and adult frogs was carried out in accordance with approved guidelines and was performed with minimal or non-invasive techniques, respectively. Animals were released immediately after sampling. Procedures were approved by the local ethics committee for animal experiments (2. Lokalna Komisja Etyczna do Spraw Doświadczeń na Zwierzętach, Wrocław, permit no. 54/2013).

### Mitotyping and genotyping

We inferred the mitochondrial haplogroups of 565 samples using a mitotyping procedure by restriction digest of *cytochrome-b* PCR products (described in ref.^45^). We genotyped 352 individuals from the contact zone for 29 microsatellite loci mapped throughout the genome in *H. arborea*, including 8 sex-linked and 21 autosomal^67^, cross-amplifying in both species^68^. Sex-linked markers cover the entire sex chromosome, as inferred from linkage mapping analyses^48^,^67^. Microsatellites were amplified in multiplexes^68^; amplicons were run on an ABI3100 genetic analyzer and scored with Genemapper 4.0 (Applied Biosystems). Details of markers and references are available in File S4 and have been archived at http://doi:10.5061/dryad.5jq64.

### Population genetic analyses

In order to locate hybrid populations and document patterns of introgression between *H. arborea* and *H. orientalis*, we characterized the genetic structure throughout the study area using STRUCTURE^69^. We used the admixture model without prior on sample origin and tested from one to 11 groups (K) with 10 replicate runs per K, each run consisting of 100,000 iterations following a burn-in period of 10,000. The Evanno method^70^, implemented in STRUCTURE HARVESTER^71^, suggests K is not higher than 2. Replicates were combined with CLUMPP^72^ and graphical displays of ancestry coefficients (barplots) were built with DISTRUCT^73^. To confirm that the two tree frog lineages are genetically structured (i.e. K ≠ 1, which cannot be tested with the Evanno method), we performed a principal component analysis on individual microsatellite genotypes (R package *adegenet*)^74^. As the markers are highly informative in distinguishing between the two species (Results, see also refs^45^,^68^), the STRUCTURE coefficient of ancestry at K = 2 is a good estimator of the hybrid index (0: pure *H. orientalis*; 1: pure *H. arborea*). Individuals were considered as “confirmed” nuclear hybrids only if the 90% credible intervals (CIs) of their ancestry coefficient neither reached 0 nor 1. This conservative approach allows confidently assigned individuals to be distinguished from those with uninformative genotypes^75^.

To infer whether sex-chromosomes introgressed differentially as compared to the rest of the genome, we sampled the STRUCTURE ancestry posterior at each locus using the *site-by-site* output option, and computed average HI estimates for sex-linked and autosomal markers, respectively, for comparison with the global HI estimate. Where the geographic pattern of hybridization is complex, Szymura & Barton^28^ suggest comparison of introgressing loci to the overall hybrid index rather than geographic distance^76^. If introgression is homogeneous over the genome the expectation for the hybrid index at any subset of the markers is the same as the global estimate^28^. To assess whether this is the case at sex-linked *vs* autosomal marker subsets, we fitted 2-parameter genomic clines^51^ and tested whether a single cline can explain all marker types (2-parameters model) or two separate clines, one for each marker type (4-parameters model). Moreover, in “confirmed” hybrid individuals, differential introgression between marker sets was tested using non-parametric paired tests.

Since STRUCTURE ancestry estimates depend on both the level of admixture and the informativeness of genotypes, we ran additional analyses to control for this issue. First, we re-estimated autosomal and sex-linked hybrid indices independently in separate STRUCTURE runs. This confirms that each datasets is powerful enough on its own to distinguish between the species and that their introgression signals are sufficiently distinct that the distinction remains when STRUCTURE is run for genotypes combining both marker sets. Second, to account for differences in statistical power for detecting introgression due to the different number of markers in each set (8 and 21 sex-linked and autosomal loci respectively), and to ensure the average autosomal introgression signal was not unduly influenced by a few far-introgressing outliers, we computed autosomal hybrid indices from 100 resampled datasets of 8 randomly chosen loci.

## Additional Information

**How to cite this article**: Dufresnes, C. *et al*. Empirical evidence for large X-effects in animals with undifferentiated sex chromosomes. *Sci. Rep*. **6**, 21029; doi: 10.1038/srep21029 (2016).

## Supplementary Material

Supplementary Information
