# Supplementary material for: Empirical evidence for large X-effects in animals with undifferentiated sex chromosomes
Source: Sci Rep. 2016 Feb 12;6:21029. doi: 10.1038/srep21029 (PMC4751523; doi:10.1038/srep21029)
Supplement: Supplementary Information [file srep21029-s1.pdf]

SREP21029; Supplementary Information

Title:

Empirical evidence for large X-effects in animals
with undifferentiated sex chromosomes

Authors:

Christophe Dufresnes, Tomasz Majtyka, Stuart J.E. Baird, Jörn F. Gerchen,
Amaël Borzée, Romain Savary, Maria Ogielska, Nicolas Perrin,
Matthias Stöck

File S1: Principal Component Analysis (PCA) of microsatellite genotypes.
The first axis, which explains 13.3 % of the total variance, accounts for interspecific differences between the *H. arborea* (green) and *H. orientalis* (blue) gene pools.

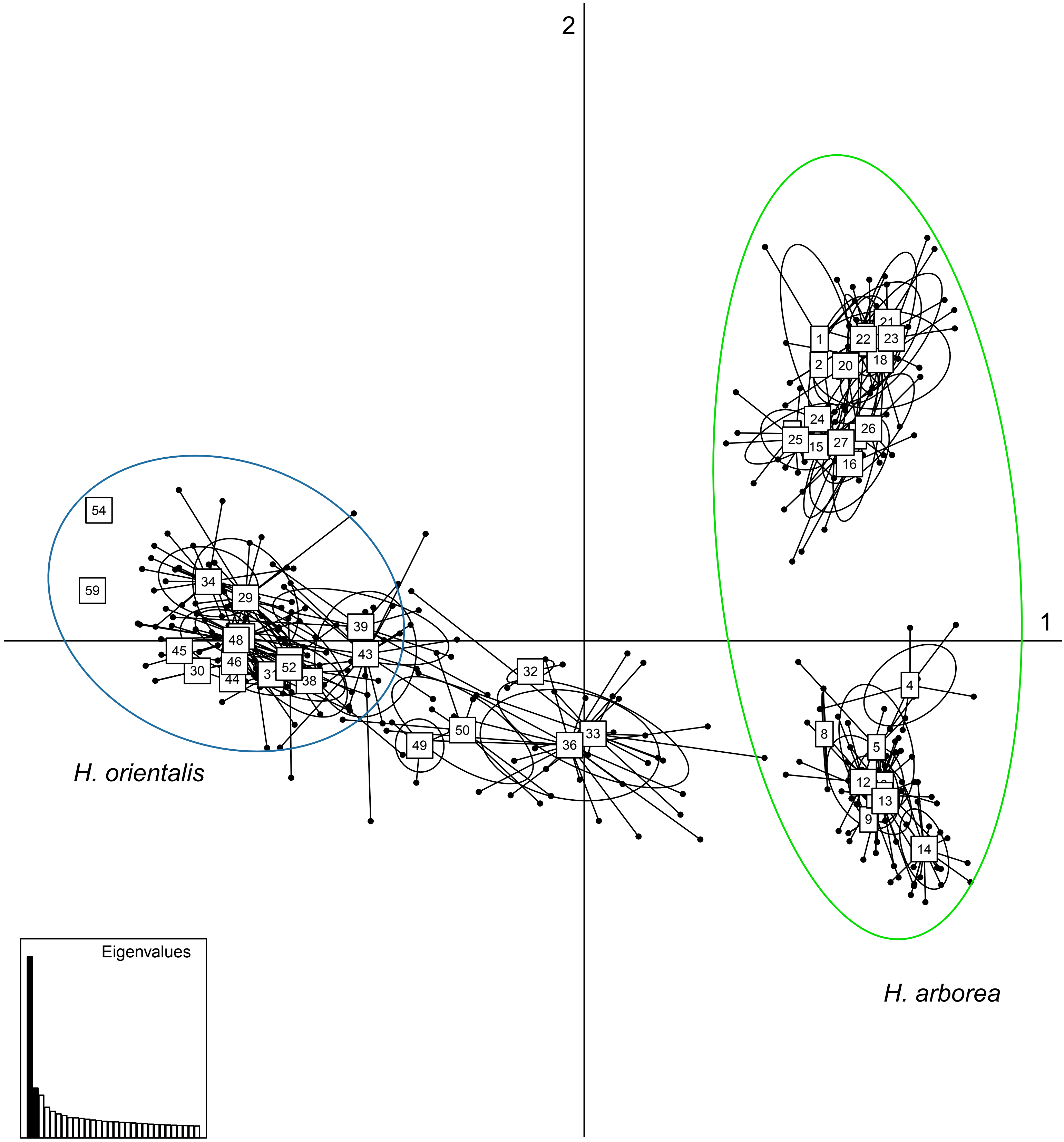

File S2: Comparison of sex-linked vs. autosomal introgression estimated independently.

(a) Autosome and sex-chromosome *H. arborea* ancestry, compared to the genome background.
(b) Differential introgression in confirmed hybrids (non-parametric Wilcoxon test, p = 2.0 e-10).
(c) STRUCTURE barplots of admixture proportions for sex-linked, autosomal and all markers in the study area.
(d) Distribution of the Wilcoxon's p-values obtained from 100 comparisons of sex-linked vs. autosomal introgression, the latter estimated from resampled datasets of 8 randomly chosen loci.

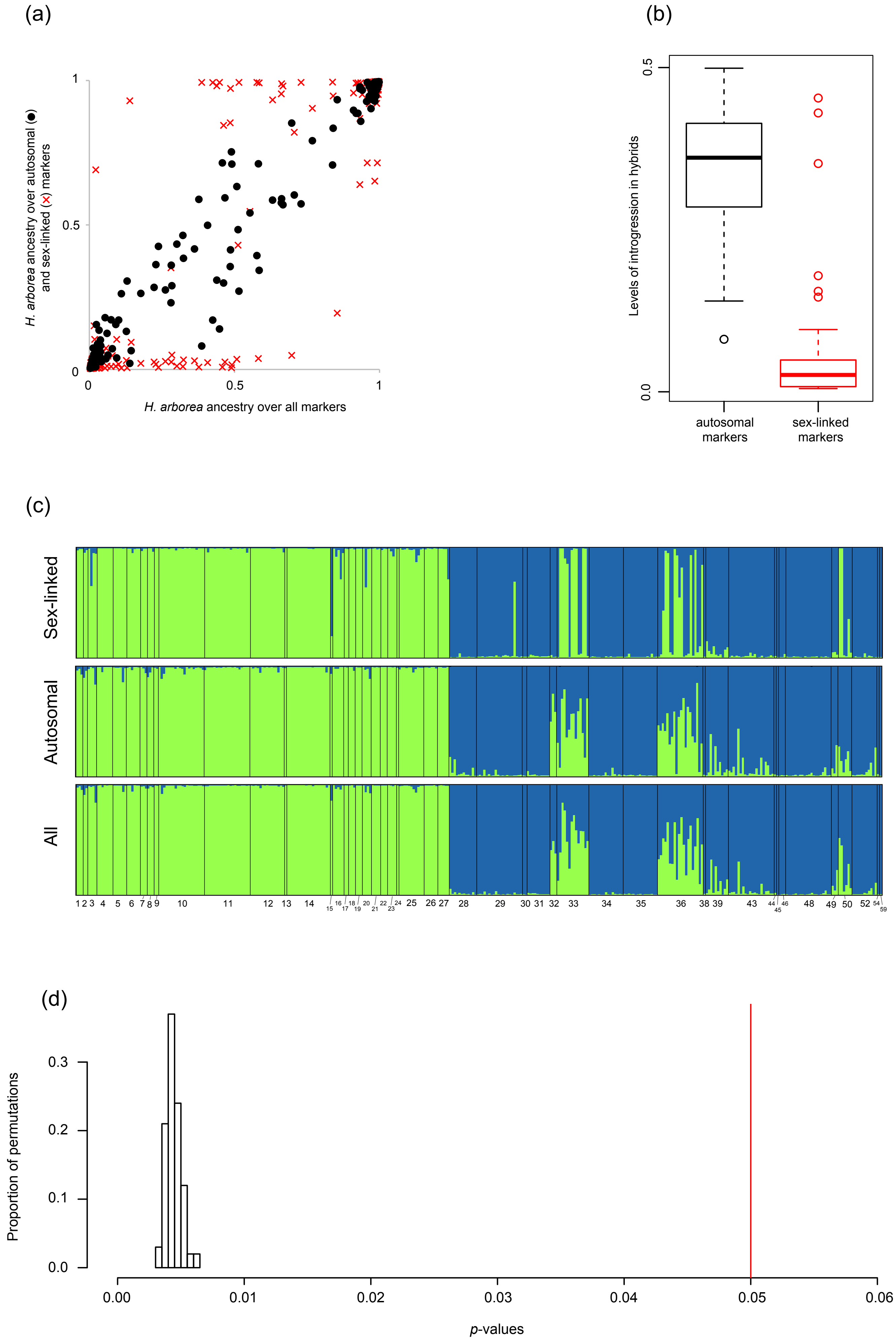

File S3: Origin of the samples used in this study (all from Poland). Sample sizes (n) are detailed by sexes (M: male, F: female, U: unknown).

ID	Locality	Latitude	Longitude	n	M	F	U
1	Zgorzelec	51.19	15.11	**3**	-	-	3
2	Nowy Raduszec	52.03	15.05	**2**	-	2	-
3	Zary	52.04	15.16	**4**	-	3	1
4	Lubrza	52.31	15.44	**7**	-	-	7
5	Rosin	52.16	15.59	**6**	-	-	6
6	Krepa	52.03	15.56	**6**	6	-	-
7	Chwalim	52.07	15.8	**3**	3	-	-
8	Szczecin	53.31	14.71	**3**	2	1	-
9	Siemidarzno	54.03	15.35	**2**	2	-	-
10	Goscino	54.06	15.63	**20**	15	5	-
11	Klepnica	53.72	15.61	**20**	20	-	-
12	Kluczewo	53.65	16.18	**15**	13	2	-
13	Szczecinek	53.74	16.66	**1**	1	-	-
14	Wytowno	54.58	16.95	**19**	19	-	-
15	Rolantowice	50.93	16.9	**1**	-	-	1
16	Pruszowice	51.19	17.14	**5**	-	-	5
17	Przemet	52.01	16.31	**2**	-	-	2
18	Wasowo	52.35	16.26	**3**	2	1	-
19	Pniewy	52.57	16.2	**3**	3	-	-
20	Wronki	52.62	16.41	**4**	4	-	-
21	Szamotuly	52.53	16.5	**4**	2	-	2
22	Dakowy Mokre	52.26	16.5	**3**	3	-	-
23	Parsko	52.01	16.63	**4**	3	1	-
24	Lusówko	52.44	16.66	**1**	1	-	-
25	Nowe Miasto nad Warta	52.09	17.38	**11**	-	-	11
26	Zielonka	52.56	17.14	**6**	6	-	-
27	Brzezno	52.64	17.1	**6**	5	1	-
28	Olesnica	52.99	16.86	**12**	12	-	-
29	Wyrzysk	53.15	17.28	**35**	6	-	29
30	Czluchów	53.66	17.33	**2**	2	-	-
31	Wystep	53.11	17.65	**10**	10	-	-
32	Galezewo	52.72	17.69	**3**	3	-	-
33	Slaboszewo	52.79	17.99	**14**	14	-	-
34	Bydgoszcz	53.17	18.19	**37**	5	-	32
35	Komorza Wielka	53.64	17.86	**15**	15	-	-
36	Skarszewy	53.96	18.39	**20**	10	10	-
37	Torun	52.96	18.72	**1**	1	-	-
38	Czamanin	52.48	18.71	**1**	1	-	-
39	Uniejów	51.96	18.79	**10**	-	-	10

40	Dzialoszyn	51.1	18.77	**21**	20	1	-
41	Wola Cyrusowa	51.9	19.4	**37**	-	-	37
42	Rogozno	51.97	19.49	**18**	-	-	18
43	Wolucza	51.84	20.29	**20**	20	-	-
44	Wiskitki	52.09	20.4	**1**	-	-	1
45	Gorzewnica	52.37	20.37	**1**	1	-	-
46	Wiaczenin	52.44	19.87	**3**	2	1	-
47	Sikórz	52.64	19.59	**8**	7	1	-
48	Gorczenica	53.21	19.37	**20**	18	2	-
49	Marwica	54.02	19.54	**3**	3	-	-
50	Eldyty Wielkie	54.01	20.15	**6**	6	-	-
51	Rychnowo	53.61	20.1	**18**	17	1	-
52	Pajtuny	53.74	20.7	**11**	11	-	-
53	Stara Wies	53.2	20.77	**18**	16	2	-
54	Ruda	52.17	21.45	**1**	1	-	-
55	Urwitalt	53.82	21.65	**20**	19	1	-
56	Lomza	53.15	22.17	**2**	2	-	-
57	Zajki	53.22	22.58	**6**	5	1	-
58	Dawidowizna	53.49	22.78	**3**	2	1	-
59	Bialowieza	52.72	23.8	**21**	18	3	-
60	Wincencin	51.39	23.26	**16**	16	-	-

File S4: References of the microsatellite markers used in this study. Location on whether sex-linked or autosomal (auto.) is indicated. For sex-linked markers, relative position along the sex chromosomes is provided.

Locus	location	GenBank	Reference
***Ha*-T3**	sex-linked - LG1 (0.0 cM)	-	Brelsford *et al.* 2013, *Evolution* 67:2434-2440
***Ha*-T52**	sex-linked - LG1 (1.7 cM)	-	Brelsford *et al.* 2013, *Evolution* 67:2434-2440
***Ha*-T51**	sex-linked - LG1 (25.0 cM)	-	Brelsford *et al.* 2013, *Evolution* 67:2434-2440
***Ha*-T11**	sex-linked - LG1 (~60 cM)	-	Brelsford *et al.* 2013, *Evolution* 67:2434-2440
***Ha*-T45**	sex-linked - LG1 (prob. between *Ha*-T11 and WHA1-60)	-	Brelsford *et al.* 2013, *Evolution* 67:2434-2440
**WHA1-60**	sex-linked - LG1 (110.2 cM)	AJ403989	Arens *et al.* 2000, *Mol Ecol* 9:1944-1946
**WHA5-22**	sex-linked - LG1 (124.2 cM)	AJ403996	Arens *et al.* 2000, *Mol Ecol* 9:1944-1946
***Ha*-H108**	sex-linked - LG1 (163.7 cM)	EU029102	Berset-Brändli *et al.* 2008, *Mol Ecol Resour* 8:1095-1097
***Ha*-A130**	auto. - LG2	EU029097	Berset-Brändli *et al.* 2008, *Mol Ecol Resour* 8:1095-1097
***Ha*-E2**	auto. - LG2	EU029103	Berset-Brändli *et al.* 2008, *Mol Ecol Resour* 8:1095-1097
***Ha*-T32**	auto. - LG4	-	Brelsford *et al.* 2013, *Evolution* 67:2434-2440
***Ha*-T41**	auto. - LG4	-	Brelsford *et al.* 2013, *Evolution* 67:2434-2440
***Ha*-T49**	auto. - LG4	-	Brelsford *et al.* 2013, *Evolution* 67:2434-2440
***Ha*-T50**	auto. - LG4	KF598777	Dufresnes *et al.* 2013, *Mol Ecol* 22:5669-5684
***Ha*-T66**	auto. - LG4	KF598783	Dufresnes *et al.* 2013, *Mol Ecol* 22:5669-5684
***Ha*-T67**	auto. - LG5	KF598786	Dufresnes *et al.* 2013, *Mol Ecol* 22:5669-5684
***Ha*-T60**	auto. - LG6	KF598780	Dufresnes *et al.* 2013, *Mol Ecol* 22:5669-5684
***Ha*-T64**	auto. - LG7	KF598782	Dufresnes *et al.* 2013, *Mol Ecol* 22:5669-5684
***Ha*-T53**	auto. - LG8	-	Dufresnes *et al.* 2014, *Mol Ecol Resour* 14:716-725
***Ha*-T58**	auto. - LG8	KF598779	Dufresnes *et al.* 2013, *Mol Ecol* 22:5669-5684
***Ha*-A127**	auto. - unl.	EU029096	Berset-Brändli *et al.* 2008, *Mol Ecol Resour* 8:1095-1097
***Ha*-B5R3**	auto. - unl.	EU029098	Berset-Brändli *et al.* 2008, *Mol Ecol Resour* 8:1095-1097
***Ha*-T56**	auto. - unl.	KF598778	Dufresnes *et al.* 2013, *Mol Ecol* 22:5669-5684
***Ha*-T63**	auto. - unl.	KF598781	Dufresnes *et al.* 2013, *Mol Ecol* 22:5669-5684
***Ha*-T68**	auto. - unl.	KF598784	Dufresnes *et al.* 2013, *Mol Ecol* 22:5669-5684
***Ha*-T54**	auto. - ?	KF895912	Dufresnes *et al.* 2014, *Mol Ecol Resour* 14:716-725
***Ha*-T55**	auto. - ?	KF895913	Dufresnes *et al.* 2014, *Mol Ecol Resour* 14:716-725
***Ha*-T61**	auto. - ?	KF895914	Dufresnes *et al.* 2014, *Mol Ecol Resour* 14:716-725
***Ha*-T69**	auto. - ?	KF598785	Dufresnes *et al.* 2013, *Mol Ecol* 22:5669-5684
